# Chronic Nasal Catarrh

**Published:** 1876-03

**Authors:** E. H. Gibbs


### *CHRONIC NASAL CATARRH.*

BY E. H. GIBBS, A.M. M.D.

The chronic form of nasal catarrh is often the result of neglect of the disease in its acute stage. It is a chronic inflammation of the nasal passages, and may vary in intensity, from a feeling of uneasiness and heat in the nostrils, to painful ulcerations and copious discharges of purulent and fetid matter, which is often revoltingly offensive. This condition can not long exist without seriously impairing the health of the person. The disease has a tendency to extend down the bronchia to the lungs—its march in that direction being favored by the impaired condition of the general health, caused partly by the incessant drain upon the system by the nasal discharges.

The causes of the long continuation of the disease are numerous; they can not be simply local, for if they were, they could be reached and overcome by local remedies. Persons of sound constitutions enjoy an almost entire immunity from chronic diseases of the air passages, though suffering often from the acute variety. In the treatment of "chronic catarrh," we must endeavor to find out the condition or diathesis of each particular case, upon which the diseased condition depends. I believe that the ill success that usually attends the efforts of physicians to treat this disease successfully may be attributed to their neglect or inability to trace the disease back to its primary cause or causes.

Although these are often obscure, they are almost always discernible to the careful and experienced eye. In our investigations we should first ascertain if there is not a local cause for the trouble, such as a foreign body lodged in the nostrils, and acting as a nucleus for the deposition of solid matters from the secretions. Failing to find any sufficient local cause, our attention should be directed to a thorough examination of the physical condition of the patient; allowing no fact—however trivial to the superficial observer—to escape our notice. Nelaton, the greatest surgeon that the world has ever seen, worked out some of his most wonderful problems in surgical diagnosis from hints too obscure to attract the notice of any other surgeon. A sufferer from catarrh may spend years in torturing himself with "sea salt," carbolic acid, nitrate of silver, etc., etc., to no purpose, and finally be cured in thirty days by applying the proper remedies, not to the nasal passages, but to some weak point in the system, upon which his catarrh depended. A tendency to scrofula is, no doubt, the most usual cause of this affection. Whatever taint exists in the system, whether scrofulous, gouty, or any other, is an element of weakness which always stands in the way of recovery, and must be removed as a preliminary to any treatment. We find, therefore, that the surest and most speedy way of curing chronic catarrh is to improve the general health first, and apply the local treatment afterward. A strict compliance with these conditions, will usually result in a cure.

---

### *NOTICE.*

Our reserved copies of Hall's Journal of Health for November, 1875, and for January and February, 1876, have been exhausted by the unprecedented demand, and we would like about 1,000 copies of the November, 1875, number, and 1,500 copies of January and February, 1876. We will give any two future numbers for every one of the above described numbers delivered to us, or sent to us by mail.

---

### *THE HEALTH LIFT.*

We must not forget to keep our promise to speak a few words about this delightful system of exercise. A lift, gradually increased in amount from day to day, so that growth of muscular power is secured, proves decidedly advantageous, especially in cases of torpid digestion. We are continuing the practice, at the rooms No. 46 East Fourteenth Street, with as much regularity as is possible for a very busy person, and enjoy it greatly. There is a remarkable degree of elasticity and warmth imparted by the use of the Reactionary Lifting Machine devised by Rev. Mr. Mann, and we have no doubt the world is being made a good deal better and stronger for its daily use.

